# Evaluation of Prebiotic Potential of Three Marine Algae Oligosaccharides from Enzymatic Hydrolysis

**DOI:** 10.3390/md17030173

**Published:** 2019-03-18

**Authors:** Zhen-Lian Han, Min Yang, Xiao-Dan Fu, Meng Chen, Qian Su, Yuan-Hui Zhao, Hai-Jin Mou

**Affiliations:** College of Food Science & Engineering, Ocean University of China, 5 Yushan Road, Qingdao 266003, China; ahanzhenlian@163.com (Z.-L.H.); minyang89@163.com (M.Y.); luna_9303@163.com (X.-D.F.); meng18356975575@163.com (M.C.); suqian0533@163.com (Q.S.)

**Keywords:** algae oligosaccharides, SCFA, fecal microbiota, butyrate-producing bacteria

## Abstract

Alginate oligosaccharides (AlgO), agarose oligosaccharides (AO), and κ-carrageenan oligosaccharides (KCO) were obtained by specific enzymatic hydrolysis method. The molecular weight distributions of the three oligosaccharides were 1.0–5.0 kDa, 0.4–1.4 kDa, and 1.0–7.0 kDa, respectively. The culture medium was supplemented with the three oligosaccharides and fermented by pig fecal microbiota in vitro, for 24 h. Each oligosaccharide was capable of increasing the concentration of short-chain fatty acids (SCFAs), especially butyric acid, and altering the microbiota composition. Linear discriminant analysis effect size (LEfSe) analysis results showed that the opportunistic pathogenic bacteria *Escherichia*, *Shigella*, and *Peptoniphilus*, were significantly decreased in AlgO supplemented medium. AO could improve the gut microbiota composition by enriching the abundance of Ruminococcaceae, *Coprococcus*, *Roseburia*, and *Faecalibacterium*. Besides, KCO could increase the abundance of SCFA microbial producers and opportunistic pathogenic flora. Therefore, these results indicate that AlgO and AO can be used as gut microbial regulators and can potentially improve animal/human gastrointestinal health and prevent gut disease, whereas the physiological function of KCO needs further evaluation.

## 1. Introduction

Non-digestible oligosaccharides (NDOs) are a series of low molecular weight carbohydrates, either naturally occurring, obtained from hydrolysis of polysaccharides, or by chemical synthesis from disaccharides [1]. Accumulating evidence indicates that some NDOs (also called functional oligosaccharides) offer a significant benefit to the host health by stimulating the growth of beneficial bacteria and modulating the composition of colonic microbiota, thereby meeting the criterion of prebiotics [2,3]. Therefore, an understanding of the mechanism of interaction between oligosaccharides, intestinal microbiota, and bacterial metabolites could help to improve the gut microbiota composition, in order to promote health and prevent disease [4]. The functional oligosaccharides mainly include fructooligosaccharides (FOS), soybean meal oligosaccharides (SMO), mannan oligosaccharides (MOS), and pectin-derived acidic oligosaccharides (pAOS), which are usually found in land vegetables, fruits and soybean [5].

Seaweeds have been reported to possess higher productivity rates than terrestrial plants [6]. Seaweeds are classified into green algae, red algae, and brown algae, based on their pigmentation. Marine algae are recognized as rich resources of bioactive compounds, such as polysaccharides, proteins, lipids, and polyphenols, showing antibacterial, antiviral, and anti-inflammatory properties [7]. Additionally, studies reported that seaweed-derived poly- and oligosaccharides were also potential sources of the prebiotics [8,9]. Therefore, it is essential to further explore the applications of the algae oligosaccharides in treatment of human and animal gut health.

Alginate, agarose, and carrageenan, three main kinds of phycocolloids produced from brown algae and red algae [10], can be fermented by the gut microbiota but not digested by animal enzymes [11,12,13]. Alginate oligosaccharide (AlgO), a type of anionic oligosaccharide, is enzymatically hydrolyzed from alginate and possesses prebiotic properties, which have been shown to stimulate the growth of Bifidobacteria, in both in vitro and in vivo studies [14]. In addition, AlgO can produce gas and short-chain fatty acids (SCFAs), mainly acetic acid, propionic acid, and butyric acid, when incubated with human fecal inocula [15]. Agarose oligosaccharides (AO) from enzymatic hydrolysis of agarose also exhibit prebiotic effects, by stimulating the growth of Bifidobacteria and Lactobacilli and escaping digestion in the upper gastrointestinal tract [16]. Experts stated that the prebiotic concept of the beneficial bacterial should be widened beyond *Bifidobacterium* and *Lactobacillus*. Other beneficial bacterias such as *Ruminococcus bromii*, *Roseburia intestinalis*, *Eubacterium rectale*, and *Faecalibactrium prausnitzii* should also be taken into account [3,17]. Moreover, the concentration of SCFAs, especially of butyric acid, should be included in the prebiotic index. Therefore, although some favorable changes in gut microbiota have been evidenced in the presence of AlgO and AO, the overall structure and abundance of microbial communities and the production of SCFAs after AlgO and AO addition remain unclear. Previous studies also showed that κ-carrageenan oligosaccharides (KCO) exhibited antioxidant [18] and both antitumor effects [19,20]. However, to our current knowledge, studies on the of prebiotic property of KCO are sparse.

Since pig digestive tracts are highly similar to humans with respect to their anatomy and physiology, they are used as a model for the preliminary investigation on the metabolism of the digestive system [21]. In the present study, high-throughput sequencing of the V3–V4 regions of 16S rRNA was utilized to study the pig fecal bacterial population. We evaluated the in vitro fermentation with pig fecal microbiota of three oligosaccharides (AlgO, AO, and KCO) prepared by the enzymatic hydrolysis method, and attempted to understand (i) their fermentability by pig fecal microbiota; (ii) their fermentability associated with the production of SCFAs; and (iii) the effect of oligosaccharides on the composition of bacterial microbiota and community structure.

## 2. Results

### 2.1. Utilization of AlgO, AO, and *KCO*

AlgO, AO and KCO were prepared by enzymatic hydrolysis of alginate, agarose, and κ-carrageenan, respectively. The molecular weight distributions of AlgO, AO, and KCO were examined by high-performance size-exclusion chromatography (HPSEC) (Figure S1). The molecular weight distribution of AlgO ranged between 1.0 and 5.0 kDa. The molecular weight distribution of AO ranged between 0.4 and 1.4 kDa. The molecular weight distribution of KCO ranged between 1.0 and 7.0 kDa.

**Figure 1:**
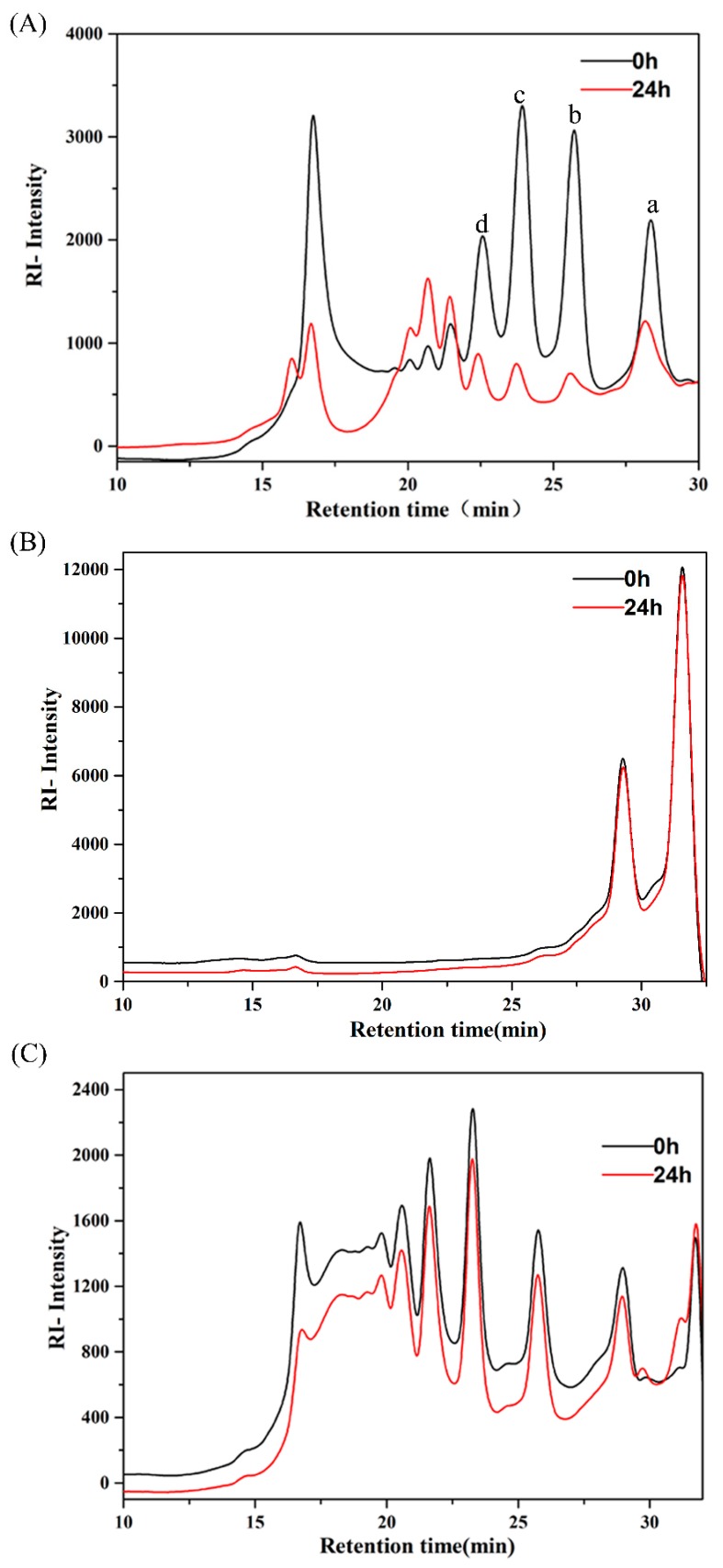
High-performance size-exclusion chromatography (HPSEC) elution patterns of oligosaccharides used in the experiment. Molecular weight distributions of alginate oligosaccharides (AlgO) (**A**), agarose oligosaccharides (AO) (**B**), and κ-carrageenan oligosaccharides (KCO) (**C**) were monitored before and after in vitro fermentation using fecal inoculum from pigs. a, b, c, and d in Figure 1A stand for disaccharides, trisaccharides, tetrasccharides, and pentasaccharides, respectively, which were tested by HPSEC with a column of Superdex^TM^ Increase 10/300 GL.

In order to assess the specific molecular weight changes of three oligosaccharides after 24 h fermentation, the AlgO, AO, and KCO degradation products were monitored by HPSEC. In the case of AlgO (Figure 1A), at 0 h, a large number of oligomers (22–30 min) and larger molecular weight (16–18 min) were observed. After 24 h fermentation, an obvious decline in the amount of oligomers, such as disaccharides, trisaccharides, tetrasccharides, and pentasaccharides, was observed and larger molecules were found to be degraded into smaller oligomers (20–22 min). In case of KCO (Figure 1C), at 0 h, a large number of oligomers (20–32 min) and larger molecules (15–20 min) were observed. A mild decrease in the number of larger molecules (15–20 min) and mild increase of smaller molecules (32 min) were observed after 24 h fermentation by pig fecal bacteria. However, AO (Figure 1B) was utilized to a smaller extent by the pig fecal microbiota after 24 h fermentation. This result indicated that AlgO was utilized and degraded by the pig fecal microbiota after 24 h fermentation, whereas KCO was degraded into smaller molecules to a lesser extent and AO was hardly utilized by the pig fecal microbiota after 24 h fermentation. 

### 2.2. SCFA Production from the Fermentation

The concentrations of SCFAs produced after 24 h fermentation in cultivation are in Table 1. The initial pH of this batch fermentation was 8.2, which decreased with the SCFA production. Acetic acid, propionic acid, butyric acid, isobutyric acid, isovaleric acid, valeric acid, and caproic acid were the SCFAs examined in all treatments. The total SCFA concentration was calculated as the sum of these seven SCFAs. In the CN group, the total concentration of SCFAs was 6.90 ± 0.83 mM. In the experiments with medium supplemented with AlgO, AO, and KCO, the total concentration of SCFAs was 8.08 ± 0.60 mM, 11.33 ± 2.39 mM, and 8.04 ± 0.26 mM, respectively, which was much higher than that in CN group. Besides, the experiments with the three oligosaccharides showed a significant (*p* < 0.01) increase in the production of butyric acid compared with CN group. Notably, the fermentation culture with AO also had a significant (*p* < 0.01) increase in the production of acetic acid, isobutyric acid, and isovaleric acid compared with CN group.

### 2.3. Change of the Fecal Microbiota Structure after the Fermentation of Oligosaccharides

**Figure 2:**
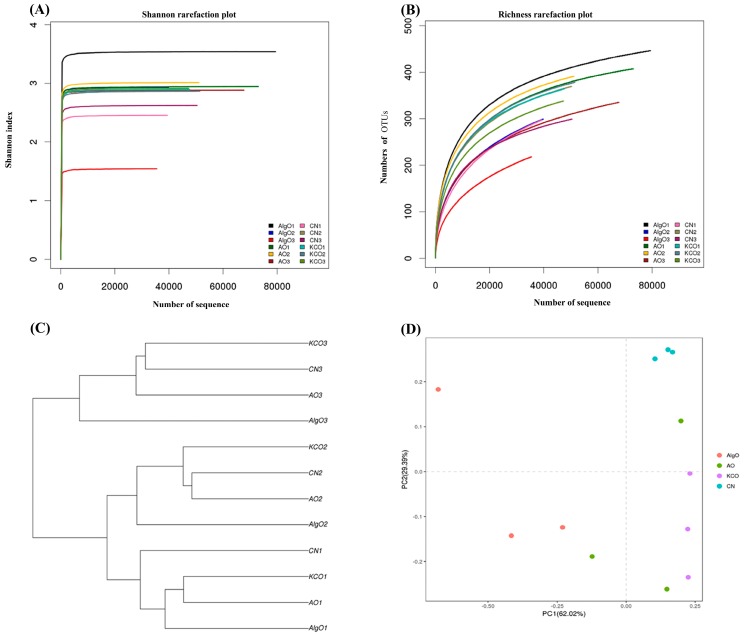
Response of the gut microbiota structure in each culture after 24 h fermentation (**A**) Shannon rarefaction analysis, (**B**) richness rarefaction analysis, (**C**) unweighted clustering analysis of gut microbiota, and (**D**) PCA score plot of gut microbiota at different OTU levels. AlgO: alginate oligosaccharides; AO: agarose oligosaccharides; KCO: κ-carrageenan oligosaccharides; CN: control.

The Shannon rarefaction analysis (Figure 2A), richness rarefaction analysis (Figure 2B), clustering analysis (Figure 2C), and principle component analysis (Figure 2D) were used to provide an overview of the changes in fecal bacteria flora after 24 h fermentation. Community diversity was assessed through the Shannon Index and the Richness Curve. By the Shannon Index and Richness Curve analysis, a large distance between the CN and AlgO groups was observed, whereas, the AO and KCO groups were in between. The principal component analysis (PCA) scores clearly separated the AlgO from the CN, both along the PC1 and PC2. Besides, the cluster analysis also indicated that the microbiota changed after AlgO treatment, especially the NO.2 and NO.3 pigs. However, AO and KCO had no obvious effect on the microbiota after 24 h fermentation. 

**Figure 3:**
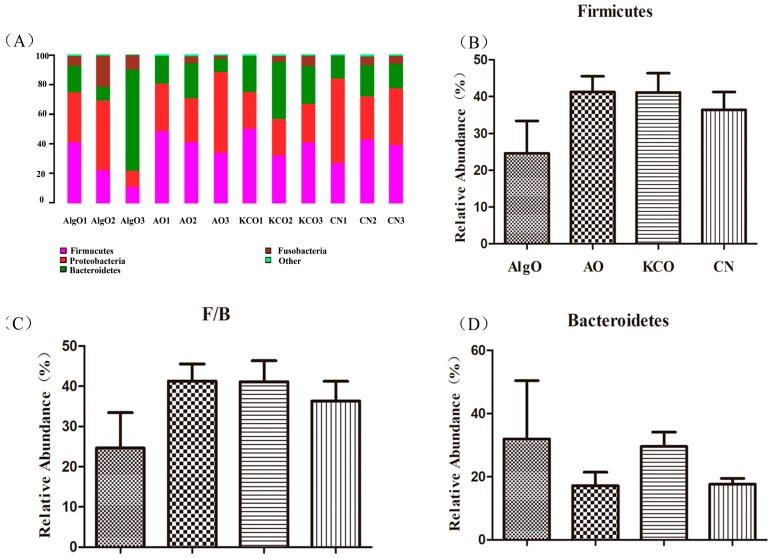
Relative abundance of different phyla in the pig fecal microbiota in each group after 24 h fermentation. (**A**) Relative abundance of gut microbiota at the phylum level, (**B**) relative abundance of Firmicutes, (**C**) relative abundance of Bacteroidetes, and (**D**) the ratio of Firmicutes and Bacteroidetes. AlgO: alginate oligosaccharides; AO: agarose oligosaccharides; KCO: κ-carrageenan oligosaccharides; CN: control; F: Firmicutes; B: Bacteroidetes.

**Figure 4:**
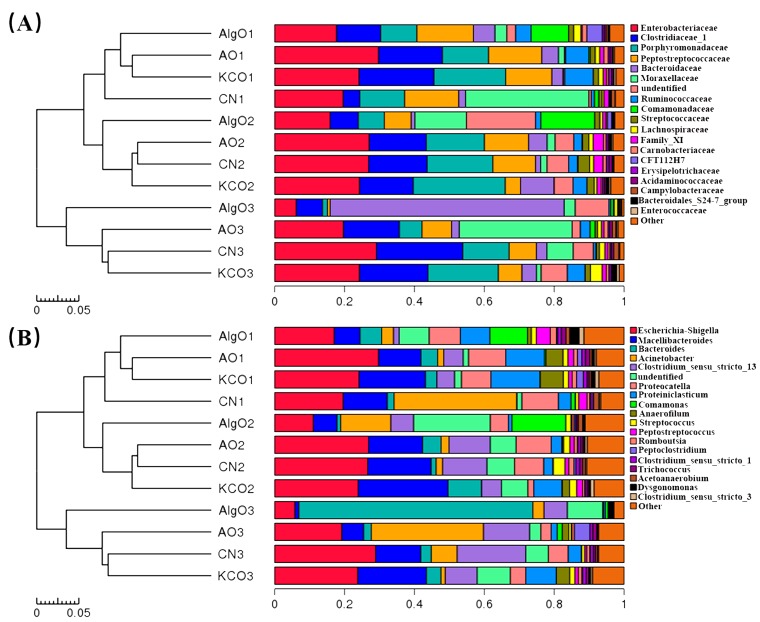
Relative abundance of gut microbiota at the family level (**A**) and genus level (**B**). AlgO: alginate oligosaccharides; AO: agarose oligosaccharides; KCO: κ-carrageenan oligosaccharides; CN: control.

To further understand the structural changes in the bacterial microbiota after being cultivated with AlgO, AO, and KCO, the bacterial populations at phylum level were assigned by the RDP classifier. The three dominant phyla were Firmicutes, Bacteroidetes, and Proteobacteria, as shown in Figure 3A. Compared to the CN group (Figure 3B–D), all the three oligosacchrides had different effects on the abundance of Bacteroidetes and Firmicutes, of which, AlgO appeared to greatly increase the abundance of Bacteroidetes and decrease the abundance of Firmicutes and Firmicutes/Bacteroidetes ratio (F/B). However, AO had no obvious effect on the abundance of Bacteroidetes and a mild increase on the abundance of Firmicutes. In KCO culture, a mild increase in the abundance of the Bacteroidetes was observed. Bacteria taxa information (genera, families, and phyla heatmap) are displayed in Figure 4A,B and Figure S2, separately.

The above results demonstrated that AlgO shifted the overall structure of pig fecal microbiota after 24 h fermentation, compared to the CN group.

### 2.4. Key Phylotypes of Fecal Microbiota Modulated by the Oligosaccharides

**Figure 5:**
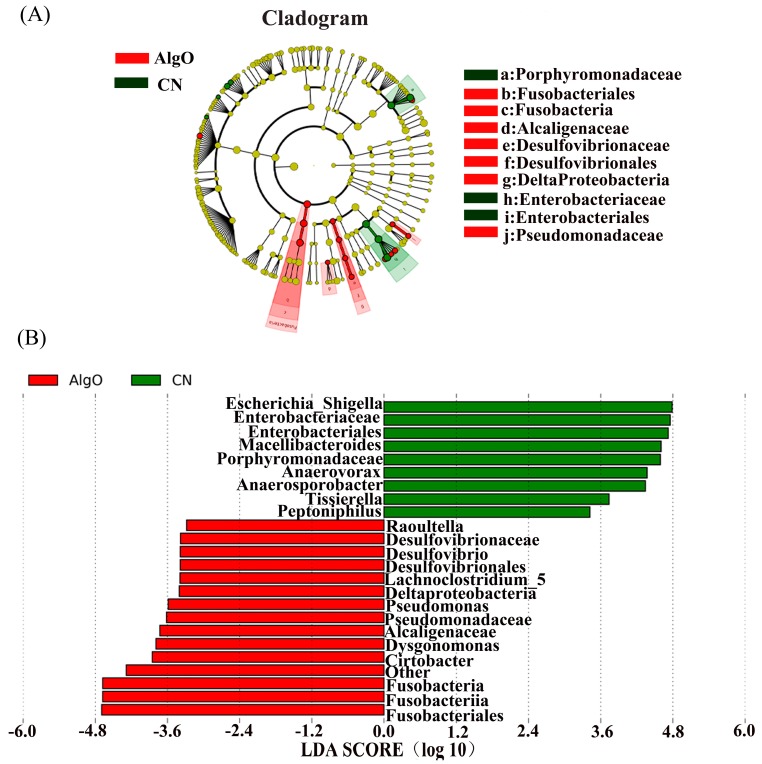
Linear discriminant analysis effect size (LEfSe) analysis identifying the significantly abundant taxon between control (CN) and the alginate oligosaccharides (AlgO) groups. (**A**) The taxa meeting a significant LDA threshold value of >2 were shown in AlgO and CN groups. (**B**) Taxonomic cladogram obtained using LEfSe analysis of the 16S sequence. Red: AlgO-enriched taxa; green: CN-enriched taxa.

**Figure 6:**
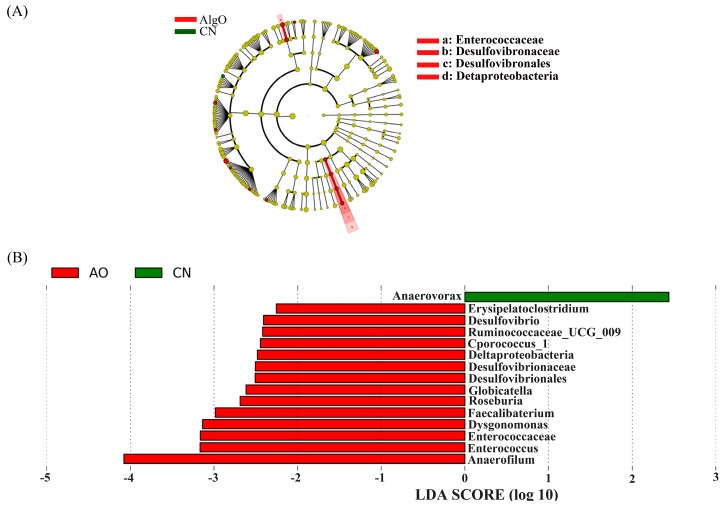
Linear discriminant analysis effect size (LEfSe) analysis identifying the significantly abundant taxon between control (CN) and agarose oligosaccharides (AO) group. (**A**) The taxa meeting a significant LDA threshold value of >2 were shown in AO and CN groups. (**B**) Taxonomic cladogram obtained using LEfSe analysis of the 16S sequence. Red: AO-enriched taxa; green: CN-enriched taxa.

**Figure 7:**
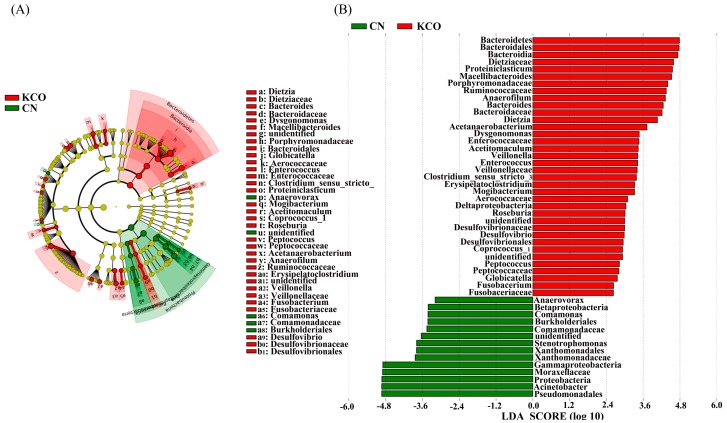
Linear discriminant analysis effect size (LEfSe) analysis identifying the significantly abundant taxon between control (CN) and κ-carrageenan oligosaccharides (KCO) groups. (**A**) The taxa meeting a significant LDA threshold value of >2 were shown in KCO and CN groups. (**B**) Taxonomic cladogram obtained using LEfSe analysis of the 16S sequence. Red: KCO-enriched taxa; green: CN-enriched taxa.

In order to assess the specific and characteristic changes in the bacterial population in each group after 24 h fermentation, linear discriminant analysis effect size (LEfSe) analysis was applied with an LDA score log_10_ > 2. A total of 24 taxa displayed a significant difference in their abundance between the AlgO and CN group (Figure 5A,B). It was worth noting that the relative abundance of *Escherichia*, *Shigella*, and *Peptoniphilus* were significantly decreased compared to the CN group. In addition, the relative abundance of *Lachnoclostridium_5* and *Pseudomonas*, *Dysgonomonas,* and *Citrobacter* were significantly increased. A total of 24 taxa displayed a significant difference in their abundance between the AO and CN groups (Figure 6A,B). The relative abundance of *Anaerovorax* was significantly decreased. In addition, the relative abundance of *Ruminococcaceae_UGG_009*, *Coprococcus_1*, *Roseburia*, *Faecalibacterium*, *Dysgonomonas*, Enterococcaceae, *Enterococcus,* and *Anaerofilum* were significantly increased by AO. Compared to the AlgO and AO group, a total of 50 taxa displayed a significant difference in their abundance between the KCO and CN group (Figure 7). Eight of the 50 taxa are shown in Figure S3A–H. The relative abundance of *Bacteroides*, *Enterococcus*, Enterococcaceae, *Coprococcus_1*, Ruminococcaceae, *Roseburia*, *Peptococcus,* and *Vellionella* were significantly increased. 

### 2.5. Link Between Microbiota Composition, SCFAs, and Three Oligosaccharides

**Figure 8:**
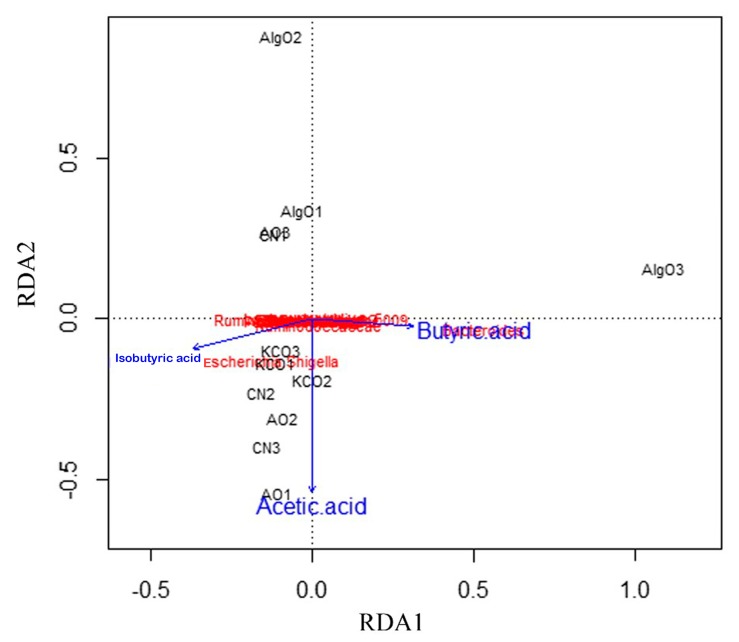
Redundancy analysis (RDA) analysis of microbiota composition and SCFAs between three oligosaccharides. Correlation plots based on an RDA depicting the relationship between microbiota composition and the differences induced by oligosaccharides. *Clostridium_sensu_stricto_1*, *Lachnoclostridium_5*, *Lachnoclostridium*, *Ruminococcaceae_UCG-013*, *Escherichia-Shigella*, *Veillonella*, *Bacteroides*, *Coprococcus_1*, *Tissierella*, *Peptococcus*, *Raoultella*, *Desulfovibrio*, *Ruminococcaceae_UCG-009*, *Enterococcus*, *Peptostreptococcus*, *Faecalibacterium* and *Anaerovorax* were subjected to redundancy analysis. AlgO: alginate oligosaccharides; AO: agarose oligosaccharides; KCO: κ-carrageenan oligosaccharides; CN: control.

To relate the changes in microbiota composition to the different oligosaccharides and different SCFAs as main metabolites of microbial fermentation, the relative abundance of 17 taxa was subjected to redundancy analysis (Figure 8). AlgO alone appeared to be obviously distinct from the CN group. Positive correlations were observed between the gut microbiota structural changes and presence of acetic acid, isobutyric acid, and butyric acid. In addition, *Escherichia* and *Shigella* were negatively correlated with butyric acid, whereas positive correlations were observed with isobutyric acid and acetic acid. The microbia, such as *Bacteroides*, *Enterococcus*, Enterococcaceae, *Coprococcus_1*, Ruminococcaceae, and *Roseburia* were positive correlated with butyric acid.

## 3. Discussion

### 3.1. Utilization and Influence of AlgO on Fermentation Pattern

AlgO are gaining increasing popularity due to their various physiological activities such as antifungal activity [22], ability to inhibit colonization by pathogenic bacteria (inhibits colonization of the large intestine of chickens by the pathogen *Salmonella enteritidis*) [23], and prebiotic activity in vitro [24] as well as in vivo [14]. In the study, an anaerobic fermentation system was employed to evaluate the fermentation ability and effect of AlgO on pig fecal microbiota. After 24 h fermentation, only partial utilization and degradation of AlgO was observed, which was in line with previous results [25]. A previous study reported less than 55% utilization of the alginate after 39 days of adaption to an alginate supplemented. Better utilization of AlgO by the microbiota was observed compared to the utilization of the large molecular of alginate, which was also supported by Jonathan using the feces of alginate-fed Pigs [26]. 

SCFAs, especially butyric acid, are known to regulate intestinal macrophage function via down-regulation of proinflammatory effectors [27], ameliorate mucosal inflammation [28], increase epithelial barrier function [29], and possess protective effects on colon cancer [30]. In this study, AlgO-supplemented medium was found to produce more butyric acid as compared to the CN group. A previous study has reported increased butyric acid production in the pig colonic digesta in the presence of supplemented pectin, which is also mainly composed of uronic acids [31]. Likewise, Salvador et al. also reported that the production of butyric acid was associated with the presence of uronic acids in AlgO (structure of AlgO shown in Figure S4) [32].

Firmicutes and Bacteroidetes were the predominant phyla, which was also reported in pigs [33]. We observed that AlgO supplementation resulted in a higher relative abundance of Bacteroidetes and lower abundance of Firmicutes in the pig fecal bacterial population. This is an interesting result, as a previous study reported that Bacteroidetes-rich microbiota correlated with a reduced potential of obesity in humans [34]. Moreover, we also found that AlgO supplementation altered the microbiota environment, which was consistent with the comment of dietary fiber shaping gut microbiota and affecting their function [35]. Our finding also showed that AlgO significantly increased the abundance of *Dysgonomonas*, while significantly decreased the abundance of *Escherichia*, *Shigella*, and *Peptoniphilus*. Previous studies have proposed that *Dysgonomonas* has broad antimicrobial activity [36] and was able to degrade AlgO [37]. Analogously, *Escherichia*, *Shigella*, are common causative agents of various infections, though typically causing dysentery. Inhibition of the growth of *Escherichia* and *Shigella* indicated that AlgO has the potential to treat diarrhea. Likewise, *Peptoniphilus*, is commonly considered the primary pathogen in various infections, especially in chronic wounds such as diabetic foot ulcers and chronic pressure [38]. The present results indicated that AlgO may heal damage of the intestine by inhibiting the growth of *Peptoniphilus*. The increase of relative abundance of genera *Pseudomonas*, *Dysgonomonas,* and *Citrobacter* [39] after addition of AlgO can explain the degradation of AlgO in the present study. However, we did not detect significant changes in the *Bifidobacterium* population in pig fecal bacteria, in line with a previous report [40]. In brief, the relative abundance of *Bifidobacterium* in the intestine of pigs is much lower, amounting to less than 1% of total bacteria. In addition, *Lachnoclostridium_5* belongs to the family of Lachnospiraceae that can produce butyric acid in the human gastrointestinal tract. In this study, it is worth noting that the presence of *Lachnoclostridium_5* was positively correlated with butyric acid production.

Therefore, our findings support the hypothesis that AlgO supplementation could alter the microbiota community to a healthier pattern. The study also provides evidence that AlgO inhibits the growth of pathogenic bacteria such as *Eschericiha*, *Shigella* and *Peptoniphilus* and has the potential to favorably aid in bodyweight management and improve the gut health in humans and animals. 

### 3.2. Utilization and Influence of AO on Fermentation Pattern

Recent evidence showed that AO exerted great prebiotic effect by stimulating the growth of Bifidobacteria and Lactobacilli, both in vitro and in vivo studies [16]. In the study, after 24 h fermentation, little degradation of AO was observed, which was also observed by previous study [41]. In brief, the degradation rate and utilization of AO maybe depends on different individuals after 24 h fermentation. 

Acetic acid, the most abundant SCFA in fecal and digesta sample, was reported for its benefits in improving cardiovascular health and function in DOCA-salt hypertensive mice [42]. In the present study, a significant increase was observed in the production of acetic acid and butyric acid compared to CN group after 24 h fermentation. The composition of AO is neoagarotetraose and neoagarohexaose, which both were linear oligosaccharides arranged 4-O-linked 3,6-anhydro-α-l-galactopyranose and 3-O-linked β-d-galactopyranose (repeat unit structure in Figure S5). A previous study also reported the presence of galactose in the polysaccharides could increase the production of acetic acid and butyric acid during the in vitro fermentation [43]. 

Ruminococcaceae, a family of autochthonous and benign members, primarily inhabit the colon and the inter-fold regions [44], that contains several butyrate-producing bacteria [45]. Moreover, a previous study reported a severe decrease in the abundance of members of the Ruminococcaceae family, including *Faecalibacterium* and *Anaerofilum*, in the alcohol-dependent subjects [46]. In nonalcoholic steatohepatitis patients, *Coprococcus* and *Roseburia* are decreased compared to the healthy individuals [47]. Besides, in ulcerative colitis (UC) patients, *Roseburia* and *Faecalibacterium* were significantly less abundant compared to the control and had a lower content of fecal butyric acid [48]. It is worthy to note that Ruminococcaceae, *Faecalibacterium*, *Anaerofilum*, *Coprococcus,* and *Roseburia* were increased in the presence of AO, in the present study. Likewise, Zhang et al. also reported that supplementation of neoagarotetraose could increase the abundance of SCFA microbial producers including Ruminococcaceae and *Roseburia* and modulate the microbiome composition in intense exercise induced fatigue mice [49]. These oligosaccharides are usually attacked by glycan degraders such as *Roseburia* and *Ruminococcus* [50].

Taking the above observations into account, our findings support the hypothesis that AO supplementation to increase availability of AO to the distal colon microbiota may improve the gut health by production of beneficial SCFAs and provide a new direction for gut disease.

### 3.3. Utilization and Influence of KCO on Fermentation Pattern

Previous studies have reported that KCO exhibited antioxidant potential [18] and prevented colon carcinogenesis [51], but little information of prebiotic property of KCO was assessed. In the present study, KCO was degraded by the pig fecal microbiota, with an increase in the production of butyric acid, compared to the CN group after 24 h fermentation. The production of butyric acid may also be associated with the presence of galactose in KCO (repeat unit structure in Figure S6). As expected, the butyric acid producing bacteria, such as *Coprococcus_1*, Ruminococcaceae, and *Roseburia,* were increased significantly, similar to that observed in the AO group. *Coprococcus_1*, Ruminococcaceae, and *Roseburia* were the secondary degraders and the genus Bacteroides was the primary degrader, during the degradation of KCO [52]. 

These observations are very interesting when it comes to our previous result, where degraded-carrageenan, mainly composed of hexaose and octaose, could have certain anti-inflammatory effects by decreasing the release of abundant of NO, in vitro [53]. Meanwhile, in contrast, Li et al. reported that KCO, with an average molecular weight of 4.5 kDa, may induce colitis, because of the existence of KCO degrading bacteria. And this result mandates further confirmation [54]. As opportunistic pathogenic flora, members of the *Peptococcus*, *Bacteroides* sp., *Fusobacterium* sp., and *Clostridium* sp. can cause disease if they proliferate [55]. In UC patients, *Clostridium* sp., *Bacteroides* sp., and *Fusobacterium* sp. were increased compared to the healthy individuals [56]. Unexpectedly, these organisms were significantly increased in the KCO group in the present study. From a fecal microbiota perspective, our results indicated that KCO may provide extent contribution to inducing UC, but also promote the production of certain SCFAs to balance the gut microbiota. Therefore, whether the 1.0–7.0 kDa molecule of KCO improves intestinal health needs further verification. 

## 4. Materials and Methods

### 4.1. Materials

Commercial-grade polysaccharides were used in this experiment. Alginate (produced from the brown algae, *Laminaria japonica)* was purchased from Sinopharm Chemical Reagent Group Co., Ltd. (Shanghai, China). Agarose (produced from the red algae, *Gelidium amansii*) was purchased from Biosharp Enterprises (Hefei, China) and κ-Carrageenan (produced from the red algae, *Kappaphycus alvarezii*) was purchased from Dehui Enterprises (Qingdao, China). Resazurin was obtained from Macklin (Shanghai, China). Alginate lyase and agarase were both produced from marine bacterium *Microbulbifer* sp. Q7 [57,58], and κ-carrageenase was produced from *Zobellia* sp. ZM-2 [59]. All other chemicals used were of analytical grade. 

### 4.2. Preparation of AlgO, AO, and KCO

Alginate (2%) was mixed with alginate lyase (28 U/mL) and incubated at 37 °C for 6 h to prepare AlgO. The sample was centrifuged after hydrolysis and the final product was obtained through precipitation of the supernatant with four times (*v/v*) of 95% ethanol. AO was prepared by enzymatic hydrolysis of agarose method as described previously with slightly modification [57]. In brief, agarose (1%) (substrate: agarase (4 U/mL) = 1:14) was incubated at 40 °C for 7 h. Following hydrolysis, the sample was centrifuged, and the supernatant was precipitated with two times (*v/v*) of 95% ethanol. Thereafter, the supernatant was precipitated with six times (*v/v*) of 95% ethanol, as the final product. KCO was made by a method described previously [58]. The three products were freeze-dried (FD-1A-50, freezer dryer, Biocool Experimental Instrument Co., Ltd., Beijing, China) and stored for fermentation. 

### 4.3. Fecal Samples and In Vitro Fermentation

Fecal samples were collected from three healthy pigs. The pigs had not received antibiotic treatment for at least 3 months before the examination, had not consumed pre or probiotic supplements, and had no history of bowel disorders. The collected fresh fecal samples were immediately store at −80 °C before fecal slurry was prepared. Feces were diluted (20% *w/v*) with phosphate buffered saline (PBS) (0.1 M, pH 7.0) and the slurry was collected after passing the mixture through a 0.075 mm sieve. Each vessel was inoculated with 10 mL of the homogenized fecal slurry to achieve the final inoculation of 2.8%. The three oligosaccharides were mixed in an autoclaved medium to achieve a final concentration of 5 mg/L (AlgO and AO) or 3 mg/L (KCO). In parallell, the control sample was maintained without any carbohydrate addition.

In vitro anaerobic batch fermentations of the three oligosaccharides were conducted according to the reported method described with slight modification [60]. The fermentation medium contained the following constituents (per liter): 2 g of tryptone, 125 µL micromineral solution (132 g CaCl_2_·2H_2_O, 100 g MnCl_2_·4H_2_O, and 10 g CoCl_2_·6H_2_O and FeCl_3_·6H_2_O per liter), 250 mL buffer solution (4 g NH_4_CHO_3_ and 35 g NaHCO_3_), 250 mL micromineral solution (5.7 g Na_2_HPO_4_, 6.2 g KH_2_PO_4_, and 0.6 g MgSO_4_. 7H_2_O per liter distilled water), 0.4 g l-cysteine HCl, and 1 mL of resazurin solution. Thereafter, the medium was sterilized at 115 °C for 30 min. Each vial was sparged with nitrogen gas, sealed with a butyl rubber plug, and capped before sterilization. Following sterilization, Na_2_S was added in each vial to remove excess oxygen. All fermentation vials were incubated in an incubator at 37 °C for 24 h. 

### 4.4. Analysis of Oligosaccharides and SCFAs

Molecular weight of the oligosaccharide was measured by HPSEC. The Agilent 1260 high performance liquid chromatography system with the PL Aquagel-OH 30 (7.5 mm × 300 mm × 8 µm) column was used for the experiment. The system was calibrated using standard Dextran of different molecular weights (1000, 3650, 5000, and 21,000 Da) with a refractive index detector (Agilent Technologies Co. Ltd., Thuringia, Germany). An eluent of 200 mM NaNO_3_ and 10 mM NaH_2_PO_4_ was used at a flow rate of 0.6 mL/min. A column of Superdex^TM^ Increase 10/300 GL was used to assess the utilization of AlgO, AO, and KCO. The eluent used was 10 mM ammonium acetate with a flow rate of 0.5 mL/min. The sample was centrifuged at 13,800 × *g* for 5 min and the supernatant was filtered through a 0.22 µm filter. The injection volume was 20 µL.

SCFA test was performed according to the method of He et al. with slight modification [61]. Briefly, the samples used for SCFAs were filtered through a 0.22 µm filter. The filtered supernatant was injected into a gas chromatograph (GC-2014, Shimadzu, Kyoto, Japan) equipped with DBWAX-DA column (30.0 mm × 0.32 mm × 0.50 µm) and flame ionization detector (FID) (Shimadzu Co. Ltd., Kyoto, Japan) The injector and detector temperature were 250 °C. Helium was used as the carrier gas. The temperature gradient program maintained at 80 °C from the start to 2 min, 150 °C from 5.5 to 6.5 min, 180 °C from 9.5 to 10.5 min, and 230 °C from 13 to 16 min. The injection volume was 0.5 µL. Seven short-chain fatty acids (acetic acid, propionic acid, isobutyric acid, butyric acid, isovaleric acid, valeric acid, and hexanoic acid) were evaluated in this study.

### 4.5. DNA Extraction and High-Throughput Sequencing

Genomic DNA was extracted from culture samples using the E.Z.N.A ^®^ Soil DNA Kit (Omega Bio-tek, Norcross, GA, USA) according to manufacturer’s protocol. The V3–V4 hypervariable regions of the 16S rDNA gene were subjected to high-throughput sequencing at Beijing Allwegene Tech., Ltd. (Beijing, China), using the Illumina Miseq PE300 sequencing platform (Illumina, Inc., San Diego, CA, USA). The V3–V4 region of the bacteria 16S rRNA gene was amplified using universal primers: forward primer, 338F (5′-ACTCCTACGGGAGGCAGCAG-3) and reverse primer, 806R (5′-GACTACHVGGGTWTCTAAT-3′). The PCR program was as follows: 95 °C for 5 min, 25 cycles at 95 °C for 30 s, 55 °C for 30 s, and 72 °C for 30 s with a final extension of 72 °C for 10 min. PCR reactions were performed in triplicate. The total reaction volume was 25 μL, containing 2.5 μL of 10× Pyrobest Buffer, 2 μL of 2.5 mM dNTPs, 1 μL of each primer (10 μM), 0.4 U of Pyrobest DNA Polymerase (TaKaRa), and 15 ng of template DNA. The amplicon mixture was applied to the MiSeq Genome Sequencer (Illumina, San Diego, CA, USA).

Amplicons were extracted from 2% agarose gels and purified using the AxyPrep DNA Gel Extraction Kit (Axygen Biosciences, Union City, CA, USA) according to the manufacturer’s instructions and quantified using QuantiFluor™-ST (Promega, Shanghai, China). Purified amplicons were pooled in equimolar amounts and paired-end sequenced (2 × 300) on an Illumina MiSeq platform. 

### 4.6. Bioinformatics Analysis

The extraction of high-quality sequences was first performed with the QIIME package (Quantitative Insights Into Microbial Ecology). Raw sequences were selected based on sequence length, quality, primer, and tag. The raw sequences were selected, and the low-quality sequences were removed. Reads which could not be assembled were discarded. The unique sequence set was classified into operational taxonomic units (OTUs) under the threshold of 97% identity using UCLUST. Chimeric sequences were identified and removed using Usearch (version 8.0.1623). The taxonomy of each 16S rRNA gene sequence was analyzed by UCLUST against the Silva119 16S rRNA database using confidence threshold of 90%.

Community diversity was assessed through the Shannon Index and the Richness Curve using Mothur and R. PCA was applied to quantify the compositional differences between microbial communities. The heatmap was implemented by using R. LEfSe analysis was performed to observe the highly dimensional fecal microbes and find the differences between two or more groups after treatment. 

### 4.7. Statistical Analysis

All the experiments were conducted in triplicate. The results were expressed as mean ± standard deviation (SD). Each of the three oligosaccharides and a blank were fermented with fecal samples obtained from the same three pigs. Statistical analysis for SCFAs production, bacterial composition, and diversity was conducted using the one-way ANOVA in conjunction with Tukey’s HSD test (SPSS, IBM, Chicago, USA). LEfSe analysis was applied based on the ANOVA test, Wilcoxon sum-rank test, and Linear Discriminant Analysis (LDA). The threshold value of ANOVA and Wilcoxon sum-rank test was 0.05. Threshold of LDA was set as 2 in this study. Correlations in bacterial community composition and environmental variables, and redundancy analysis (RDA) were used as implemented in the R. All results were considered statistically significant at * *p* < 0.05 versus the control group (CN).

## 5. Conclusions

In conclusion, the three oligosaccharides could increase the concentration of SCFAs compared to the CN group. The overall fecal microbiota structure was altered by these oligosaccharides. AlgO exhibited the most promising effects on inhibiting the growth of pathogenic bacteria such as *Escherichia*, *Shigella,* and *Peptoniphilus*, and modifying the gut microbiota composition. Besides, enriching the population of Ruminococcaceae, *Coprococcus*, *Roseburia*, and *Faecalibacterium*, dietary AO can modulate the gut to a healthier pattern. KCO could also increase the abundance of butyric acid producing bacteria, such as *Coprococcus_1*, Ruminococcaceae, and *Roseburia*, and proliferate opportunistic pathogenic flora, including *Peptococcus*, *Clostridium,* and *Bacteroides*. Although in vitro assessment of the functionality of oligosaccharides has some limitations, including selection of medium and lack of interactions between intestinal environment and the hosts, the evidence helps point to a direction for future research and application and increase our understanding of the potential of tested oligosaccharides as prebiotics. Therefore, results of the present study may have important implications in the development of AlgO and AO as functional food ingredients with potential therapeutic utilities by manipulating the gut microbiota. 

## Figures and Tables

**Table 1:** Short chain fatty acid production in batch cultures (mmol/L fermentation cultures) containing different types of oligosaccharides (AlgO, AO, and KCO).

Substrates	pH	Total SCFA	Acetic Acid	Propionic Acid	Isobutyric Acid	Butyric Acid	Isovaleric Acid	Valeric Acid	Caproic Acid
AlgO	7.54 ± 0.33	8.08 ± 0.60	3.84 ± 0.47	1.11 ± 0.05	0.63 ± 0.02	0.79 ± 0.03 **	0.65 ± 0.01	0.53 ± 0.003 **	0.54 ± 0.003
AO	7.7 ± 0.06	11.33 ± 2.39	6.74 ± 1.93 *	1.24 ± 0.30	0.68 ± 0.02 **	0.86 ± 0.11 **	0.74 ± 0.02 **	0.53 ± 0.003 **	0.54 ± 0.004
KCO	7.64 ± 0.28	8.04 ± 0.26	3.87 ± 0.14	1.10 ± 0.06	0.62 ± 0.009	0.77 ± 0.03 **	0.63 ± 0.01	0.52 ± 0.0006	0.53 ± 0.001
CN	7.65 ± 0.04	6.90 ± 0.83	3.13 ± 0.68	0.94 ± 0.10	0.61 ± 0.01	0.54 ± 0.01	0.63 ± 0.02	0.52 ± 0.0003	0.53 ± 0.001

Results were expressed as mean ± standard deviation (*n* = 3). * stands for significant difference (*p* < 0.05) compared with the CN. ** stands for extremely significant difference (*p* < 0.01) compared with the CN. AlgO: alginate oligosaccharides; AO: agarose oligosaccharides; KCO: κ-carrageenan oligosaccharides; CN: control.

## Supplementary Materials

The following are available online at https://www.mdpi.com/1660-3397/17/3/173/s1, Figure S1: High performance size exclusion chromatography (HPSEC) elution patterns of oligosaccharides used in the experiment. Molecular weight distributions of alginate oligosaccharides (AlgO), agarose oligosaccharides (AO), and κ-Carrageenan oligosaccharides (KCO) were monitored. Figure S2: Heatmap indicating phylum-level changes among the alginate oligosaccharides (AlgO), agarose oligosaccharides (AO), κ-carrageenan oligosaccharides (KCO), and control (CN) group. Figure S3: Histogram of the different taxa relative abundances in κ-carrageenan oligosaccharides (KCO) and control (CN) group. The mean and median relative abundance of different taxa are indicated with solid and dashed lines, respectively. (A–H) were the relative abundance of *Bacteroides*, *Coprococccus_1*, *Enterococcus*, *Roseburia*, *Peptococcus*, *Veillonella*, *Fusobacterium*, and Ruminococcaceae, respectively. Figure S4: The structure of Mannuronic acid (M) and Guluronic acid (G) composition of alginate oligosaccharides (AlgO). Figure S5: The repeat unit structure of agarose oligosaccharides (AO). Figure S6: The repeat unit structure of κ-carrageenan oligosaccharides (KCO).
