# Supplementary material for: Evaluation of Prebiotic Potential of Three Marine Algae Oligosaccharides from Enzymatic Hydrolysis
Source: Mar Drugs. 2019 Mar 18;17(3):173. doi: 10.3390/md17030173 (PMC6471770; doi:10.3390/md17030173)
Supplement: Supplementary file 1 [file marinedrugs-17-00173-s001.pdf]

Supplementary Information for

Evaluation of prebiotic potential of three marine algae oligosaccharides from enzymatic hydrolysis

Zhen-Lian Han, Min Yang, Xiao-Dan Fu, Meng Chen, Qian Su, Yuan-Hui Zhao*, Hai-Jin Mou*

College of Food Science & Engineering, Ocean University of China, 5 Yushan Road, Qingdao 266003, China

* Corresponding Author:

Hai-Jin Mou; E-mail: mousun@ouc.edu.cn Tel. & Fax: +86-532-8203-2290

Yuan-Hui Zhao; E-mail: zhaoyuanhui@ouc.edu.cn Tel. & Fax: +86-532-8203-2400

List of Figures

Figure S1 High-performance size-exclusion chromatography (HPSEC) elution patterns of oligosaccharides used in the experiment. Molecular weight distributions of alginate oligosaccharides (AlgO), agarose oligosaccharides (AO), and κ-Carrageenan oligosaccharides (KCO) were monitored.

Figure S2 Heatmap indicating phylum-level changes among the alginate oligosaccharides (AlgO), agarose oligosaccharides (AO), κ-carrageenan oligosaccharides (KCO) and control (CN) group.

Figure S3 Histogram of the different taxa relative abundances in κ-carrageenan oligosaccharides (KCO) and control (CN) group. The mean and median relative abundance of different taxa are indicated with solid and dashed lines, respectively. (A)-(H) were the relative abundance of *Bacteroides*, *Coprococccus_1*, *Enterococcus*, *Roseburia*, *Peptococcus*, *Veillonella*, *Fusobacterium*, and Ruminococcaceae respectively.

Figure S4 The structure of Mannuronic acid (M) and Guluronic acid (G) composition of alginate oligosaccharides (AlgO).

Figure S5 The repeat unit structure of agarose oligosaccharides (AO).

Figure S6 The repeat unit structure of κ-carrageenan oligosaccharides (KCO).

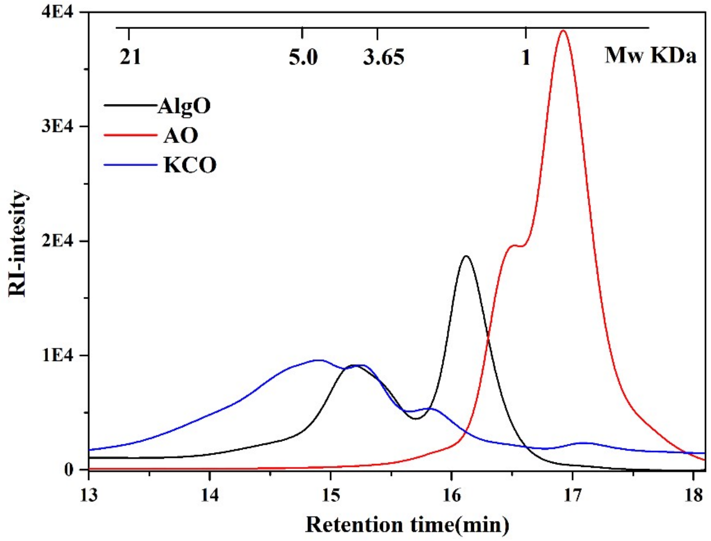

Figure S1: High-performance size-exclusion chromatography (HPSEC) elution patterns of oligosaccharides used in the experiment. Molecular weight distributions of alginate oligosaccharides (AlgO), agarose oligosaccharides (AO), and κ-Carrageenan oligosaccharides (KCO) were monitored.

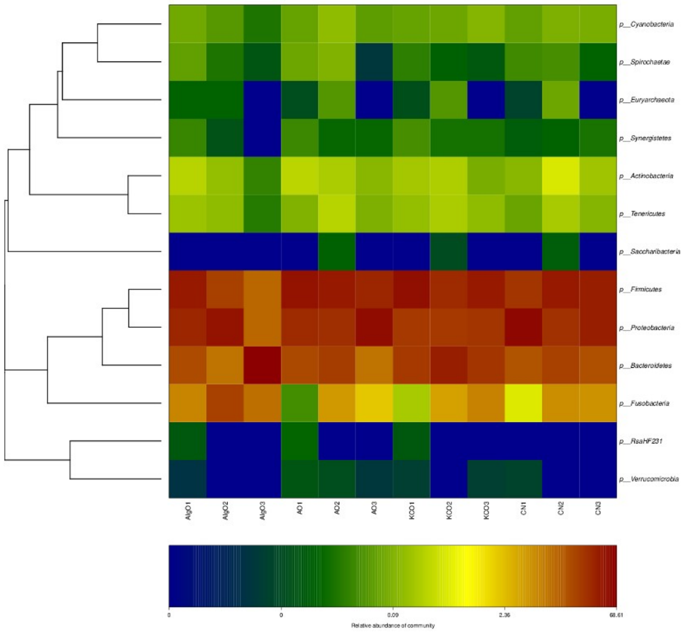

Figure S2: Heatmap indicating phylum-level changes among the alginate oligosaccharides (AlgO), agarose oligosaccharides (AO), κ-carrageenan oligosaccharides (KCO) and control (CN) group.

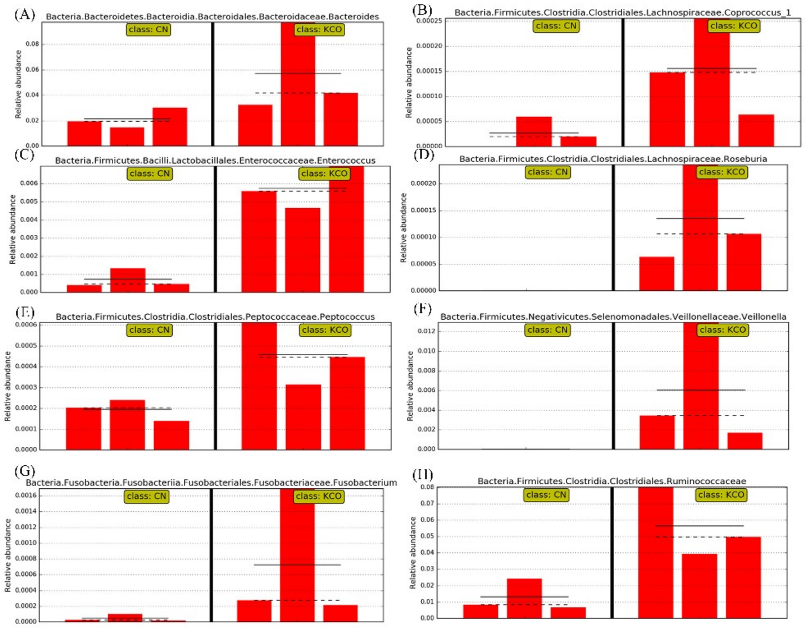

Figure S3: Histogram of the different taxa relative abundances in κ-carrageenan oligosaccharides (KCO) and control (CN) group. The mean and median relative abundance of different taxa are indicated with solid and dashed lines, respectively. (A)-(H) were the relative abundance of *Bacteroides*, *Coprococcccus_1*, *Enterococcus*, *Roseburia*, *Peptococcus*, *Veillonella*, *Fusobacterium*, and Ruminococcaceae respectively.

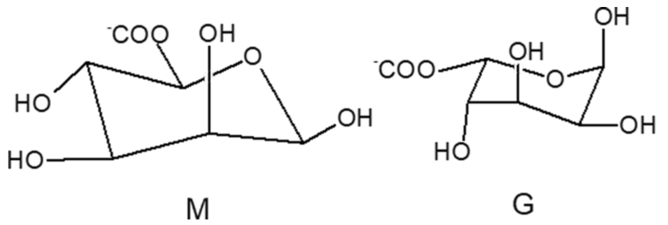

Figure S4: The structure of Mannuronic acid (M) and Guluronic acid (G) composition of alginate oligosaccharides (AlgO).

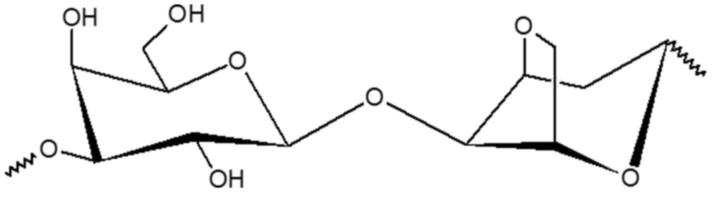

Figure S5: The repeat unit structure of agarose oligosaccharides (AO).

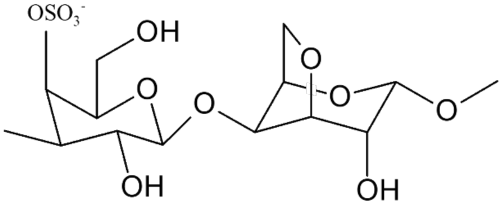

Figure S6: The repeat unit structure of κ-carrageenan oligosaccharides (KCO).
